# Predictive biomarkers of rapidly developing insulin deficiency in children with type 1 diabetes

**DOI:** 10.1136/bmjdrc-2023-003924

**Published:** 2024-02-27

**Authors:** Per Lundkvist, Annika Grönberg, Per-Ola Carlsson, Johnny Ludvigsson, Daniel Espes

**Affiliations:** 1 Department of Medical Sciences, Uppsala University, Uppsala, Sweden; 2 Department of Women's and Children's Health, Uppsala University, Uppsala, Sweden; 3 Department of Medical Cell Biology, Uppsala University, Uppsala, Sweden; 4 Department of Biomedical and Clinical Sciences, Linköping University, Crown Princess Victoria Children's Hospital and Division of Pediatrics, Linköping, Sweden; 5 Science for Life Laboratory, Department of Medical Sciences, Uppsala University, Uppsala, Sweden; 6 Science for Life Laboratory, Department of Medical Cell Biology, Uppsala University, Uppsala, Sweden

**Keywords:** Biomarkers, Children, C-Peptide, Diabetes Mellitus, Type 1

## Abstract

**Introduction:**

The rate of progression to complete insulin deficiency varies greatly in type 1 diabetes. This constitutes a challenge, especially when randomizing patients in intervention trials aiming to preserve beta cell function. This study aimed to identify biomarkers predictive of either a rapid or slow disease progression in children with new-onset type 1 diabetes.

**Research design and methods:**

A retrospective, longitudinal cohort study of children (<18 years) with type 1 diabetes (N=46) was included at diagnosis and followed until complete insulinopenia (C-peptide <0.03 nmol/L). Children were grouped into rapid progressors (n=20, loss within 30 months) and slow progressors (n=26). A sex-matched control group of healthy children (N=45) of similar age was included for comparison. Multiple biomarkers were assessed by proximity extension assay (PEA) at baseline and follow-up.

**Results:**

At baseline, rapid progressors had lower C-peptide and higher autoantibody levels than slow. Three biomarkers were higher in the rapid group: carbonic anhydrase 9, corticosteroid 11-beta-dehydrogenase isozyme 1, and tumor necrosis factor receptor superfamily member 21. In a linear mixed model, 25 proteins changed over time, irrespective of group. One protein, a coxsackievirus B–adenovirus receptor (CAR) increased over time in rapid progressors. Eighty-one proteins differed between type 1 diabetes and healthy controls. Principal component analysis could not distinguish between rapid, slow, and healthy controls.

**Conclusions:**

Despite differences in individual proteins, the combination of multiple biomarkers analyzed by PEA could not distinguish the rate of progression in children with new-onset type 1 diabetes. Only one marker was altered significantly when considering both time and group effects, namely CAR, which increased significantly over time in the rapid group. Nevertheless, we did find some markers that may be useful in predicting the decline of the C-peptide. Moreover, these could potentially be important for understanding type 1 diabetes pathogenesis.

**WHAT IS ALREADY KNOWN ON THIS TOPIC**

- Early identification of disease progression is essential for type 1 diabetes interventional trials to preserve beta cell function and improve the reliability of study findings.

**WHAT THIS STUDY ADDS**

- In this study of children with new-onset type 1 diabetes, three proteins distinguished rapid from slow progressors at baseline, and one protein, a coxsackievirus B-receptor, differed over time. Additionally, 81 proteins differed between patients with type 1 diabetes and healthy controls at baseline.

**HOW THIS STUDY MIGHT AFFECT RESEARCH, PRACTICE, OR POLICY**

- Findings can help to identify rapidly progressing type 1 diabetes when stratifying patients in interventional trials and may improve the understanding of type 1 diabetes pathogenesis.

## Introduction

Type 1 diabetes is caused by islet autoimmunity and declining insulin secretion. Environmental factors contribute to the condition^1^ in addition to genetic predisposition.^2^ Insulin depletion typically develops more rapidly in children than in adults, but the rate of progression to insulin deficiency varies greatly.

The loss of beta cell function varies with age,^3^ sex,^3^ genotype,^4^ body mass index (BMI),^5^ Haemoglobin A1c (HbA1c),^6^ as well as ketoacidosis and islet cell autoantibodies at diagnosis. A residual beta cell function carries great patient benefit as even a small preserved insulin secretion is associated with fewer episodes of ketoacidosis^7^ and serious hypoglycemia and less risk of late diabetes complications.^8^ We have recently confirmed the association between low age, ketoacidosis, higher HbA1c, high titers of glutamic acid decarboxylase antibody (GADA) and islet antigen-2 antibody (IA-2A), and a more rapid loss of endogenous insulin secretion.^9^ Moreover, we found that a rapid decline in C-peptide is associated with an increased incidence of severe hypoglycemia. Conversely, high BMI SDS (SDS=Standard deviation score, deviation of BMI from a reference population), low HbA1c during the first years, and higher frequency of the HLADR3 genotype were associated with long-term preservation of C-peptide.^9^ We have also previously found that patients with long-standing type 1 diabetes and preservation of C-peptide have increased levels of circulating interleukin (IL)-35 and a higher frequency of IL-35+ regulatory T cells (Tregs), suggestive of an altered immunological phenotype.^10^

Identifying patterns of biomarkers or individual markers that can predict disease progression rate at diagnosis could reveal novel insights regarding the pathogenesis of type 1 diabetes. Furthermore, predicting the progression rate at type 1 diabetes onset holds great value in appropriately randomizing patients to clinical intervention trials aiming to preserve beta cell function and, ideally, selecting the most appropriate beta cell preservation therapy. Therefore, we have investigated whether multiplexing of biomarkers at diagnosis can predict the rate of loss of C-peptide secretion in a cohort of children with new-onset type 1 diabetes.

## Research design and methods

### Participant selection and study design

The study is based on a retrospective, observational study including 46 children (born between 1989 and 2007) with newly diagnosed type 1 diabetes (year of diagnosis between 2004 and 2017) who initiated insulin treatment on admission. The participants were regularly followed at the Crown Princess Victoria Children’s Hospital in Linköping, Sweden. At the age of 18 years, participants were transferred to the diabetes clinic for adults. The diagnosis of type 1 diabetes was based on the criteria set by the American Diabetes Association for diagnosis and classification. At baseline, the *T1D* group was also compared with a sex-matched healthy control group of similar age, *Healthy* (N=45), from the ABIS (All Babies in Southeast Sweden) cohort.

The hypothesis was that multiplexed biomarkers can differentiate patients with rapid loss of C-peptide secretion from those with a slower decline, indicating distinct biological pathways or disease mechanisms associated with the progression of type 1 diabetes.

The aim was to investigate biomarker differences in relation to the course of residual C-peptide and whether or not multiplexed biomarkers could distinguish patients with a rapid loss of C-peptide secretion. Based on longitudinal stimulated C-peptide data, the cohort was divided into two groups, Rapid (n=20) and Slow (n=26). Rapid progression was defined as a loss of C-peptide secretion within 30 months following type 1 diabetes debut (cut-off <0.03 nmol/L). The two type 1 diabetes groups, *Rapid* and *Slow*, were also compared over time.

### Descriptive data and clinical chemistry

Descriptive data of age, sex, HbA1c, blood glucose, blood pH, and C-peptide levels at the time of diagnosis before initiating insulin treatment were collected from electronic medical records. During follow-up visits (10 days, 1, 3, 9, 18, 24, and 30 months, and 3, 4, 5, and 6 years after diagnosis), additional data were recorded, including weight, height, HbA1c, and insulin dosage (expressed as units per kilogram of body weight per 24 hours). In addition, BMI and BMI SDS, adjusted for age and sex, were automatically generated using the SWEDIABKIDS register.^11^

Mixed meal tolerance tests (MMTTs) were performed under fasting conditions in the morning. Baseline measurements of C-peptide and glucose were obtained, followed by sampling at 30-minute intervals during the 120-minute test. Short-acting insulin administration was withheld for at least 6 hours before the MMTT. The composition of the mixed meal changed over time, transitioning from a standardized breakfast to a standardized liquid meal based on the participant’s body weight. C-peptide concentrations were measured using a time-resolved fluoroimmunoassay with a lower detection limit of 0.03 nmol/L, and undetectable C-peptide levels were assigned a 0.01 nmol/L value for statistical analysis.

HbA1c and blood glucose measurements were performed at the Department of Clinical Chemistry, Linköping. The laboratory is certified by Swedac, a Swedish government authority. As of October 2010, HbA1c is analyzed using the International Federation of Clinical Chemistry and Laboratory Medicine reference method and expressed in mmol/mol. Before October 2010, analyses were based on the Mono S standard and expressed in percentage. HbA1c analyses performed with the Mono S standard were recalculated using the following expression: (International Federation of Clinical Chemistry and Laboratory Medicine (IFCC); mmol/mol) = 10.45 × HbA1c (Mono S; %)−10.62 (https://ngsp.org/convert1.asp).

Autoantibodies GADA (detection limit 5 IU/mL) and IA-2A (detection limit 7.5 kU/L) were analyzed using two-sided ELISA test kits from RSR (RSR, Cardiff, UK) in serum according to the instructions from the manufacturer. Samples negative for ELISA IA-2A were further analyzed with a high-sensitivity IA-2A radio binding assay. Recombinantglutamic acid decarboxylase 65 (GAD65) and islet antigen 2 (IA-2) were labeled with [^35^S] methionine (GE Healthcare Life Sciences, Amersham, UK) by in vitro-coupled transcription and translation in the TNT SP6 coupled reticulocyte lysate system (Promega, Southampton, UK) as described. Full-length cDNA coding for human GAD65 in the pTNT vector (Promega) (pThGAD65) or the intracellular domain (aa 603–980) of IA-2 in the pSP64 Poly(A) vector (Promega) (IA-2ic) was used. These analyses were conducted at the Department of Clinical Chemistry, Skåne University Hospital, Malmö, Sweden. The intra-assay coefficient of variation for duplicates in the GADA assay was 7% and in the IA-2A 11%. In the Diabetes Autoantibody Standardization Program 2010 workshop, our laboratory was among the top-ranking laboratories for GADA in workshop sensitivity (80%) and specificity (99%) and the top-ranking laboratory for IA-2A in workshop sensitivity (60%) and specificity (99%).^12^

### Proximity extension assay (PEA)

Protein analysis was performed in undiluted EDTA plasma by multiplex PEA at Olink Proteomics AB (Uppsala, Sweden) according to the manufacturer’s protocol. Two validated 92-plex panels, Olink IMMUNE ONCOLOGY (*IMO*) and Olink IMMUNE RESPONSE (*IRE*, online supplemental tables 1 and 2), were used to measure markers associated with inflammation and active immune and cytokine response. The IMO panel consists of proteins involved in tumor immunity, chemotaxis, tissue remodeling, apoptosis, cytotoxicity, metabolism, and autophagy. The IRE panel is focused on key proteins involved in adaptive immune response, viral defense, inflammation, and cytokine signaling. These panels were chosen based on their complementary immune profiles. Samples were collected and analyzed in patients with type 1 diabetes at 10 days, 3, 9, and 18 months after diagnosis. Additional samples were collected at 30, 48, and 72 months in those with remaining C-peptide at 30 months (Slow). Only baseline samples were analyzed in healthy controls.

10.1136/bmjdrc-2023-003924.supp1 Supplementary data

10.1136/bmjdrc-2023-003924.supp2 Supplementary data

### Data analysis and statistics

Statistical analyses were performed at Olink using R V.4.1.2 (2021-11-01), apart from baseline characteristics that were analyzed in-house using RStudio V.2022.12.0+353. Values are given as means±2 SDs. P values <0.05 after correction for multiple testing with the Benjamini and Hochberg procedure (unless otherwise stated) were considered statistically significant. Samples were analyzed in three different batches. A principal component analysis (PCA) plot was used to identify outliers among 10 bridging samples to ensure accuracy. Based on this quality control (QC) plot, the *IMO* panel had no outliers, while one baseline sample in the *IRE* panel deviated from the remaining samples. This sample was hence removed from the analysis of the *IRE* panel. All samples were included in longitudinal analyses. The samples were normalized between batches using the median difference as an adjustment factor for the limit of detection. The normalization process was evaluated using density plots and was observed to make the sample distributions more similar between batches.

PCA was further used to identify the protein patterns of each group. A Welch two-sample t-test for independent samples was used to compare differences between the two groups. Analysis of variance (ANOVA) was used to compare three groups. A linear mixed-effects regression model was used to compare the *Rapid* and *Slow* groups over time. Pearson’s χ^2^ test was used for categorical data analyses.

Applying the recommended effect size (Cohen’s f) 0.5 by Olink statistical services, a sample size of 44 in each of the two groups is required to detect differences at the 0.00027 significance level, with a power of 0.8.

## Results

### Descriptive data of study subjects

Age at onset and sex distribution did not differ between the two groups (table 1). Group *Rapid* had a higher proportion of participants with both IA-2 and glutamic acid decarboxylase (GAD) autoantibodies (88% vs 52%, p=0.04) and lower C-peptide at diagnosis than group *Slow* (0.22 vs 0.49, p=0.01). Group *Healthy* was 45 children without autoantibodies or heredity for type 1 diabetes matched for age and sex with group *T1D*.

**Table 1:** Baseline characteristics

Characteristic	Rapid (n=20)	Slow (n=26)	P value
Female n (%)	13 (65)	14 (54)	0.60
Age, years	9.96 (2.29)	10.92 (2.60)	0.20
C-peptide, nmol/L	0.22 (0.12)	0.49 (0.44)	0.01*
HbA1c, mmol/mol	103.79 (19.27)	95.81 (30.95)	0.30
HbA1c%	11.67 (1.79)	10.92 (2.84)	0.30
BMI SDS, kg/m^2^	−0.29 (1.02)	0.04 (1.41)	0.40
IA-2A+, n/N (%)	15/17 (88)	15/23 (65)	0.20
GADA+, n/N (%)	15/17 (88)	17/23 (74)	0.50
IA-2A+GADA+, n/N (%)	15/17 (88)	12/23 (52)	0.04*

Baseline characteristics by group. The proportion of IA-2 and GAD antibodies and C-peptide at baseline differed significantly between groups Rapid and Slow.

Significant *

BMI SDS, body mass index SD deviation score; GAD, glutamic acid decarboxylase; GADA, glutamic acid decarboxylase antibody.IA-2, islet antigen 2; IA-2A, islet antigen-2 antibody.

### Proximity extension assay

Twelve non-normalized samples were excluded from this visual analysis but were otherwise included. In addition, one outlier from the *IRE* panel was excluded from all analyses (online supplemental table 3).

10.1136/bmjdrc-2023-003924.supp3 Supplementary data

### Principal component analysis

**Figure 1:**
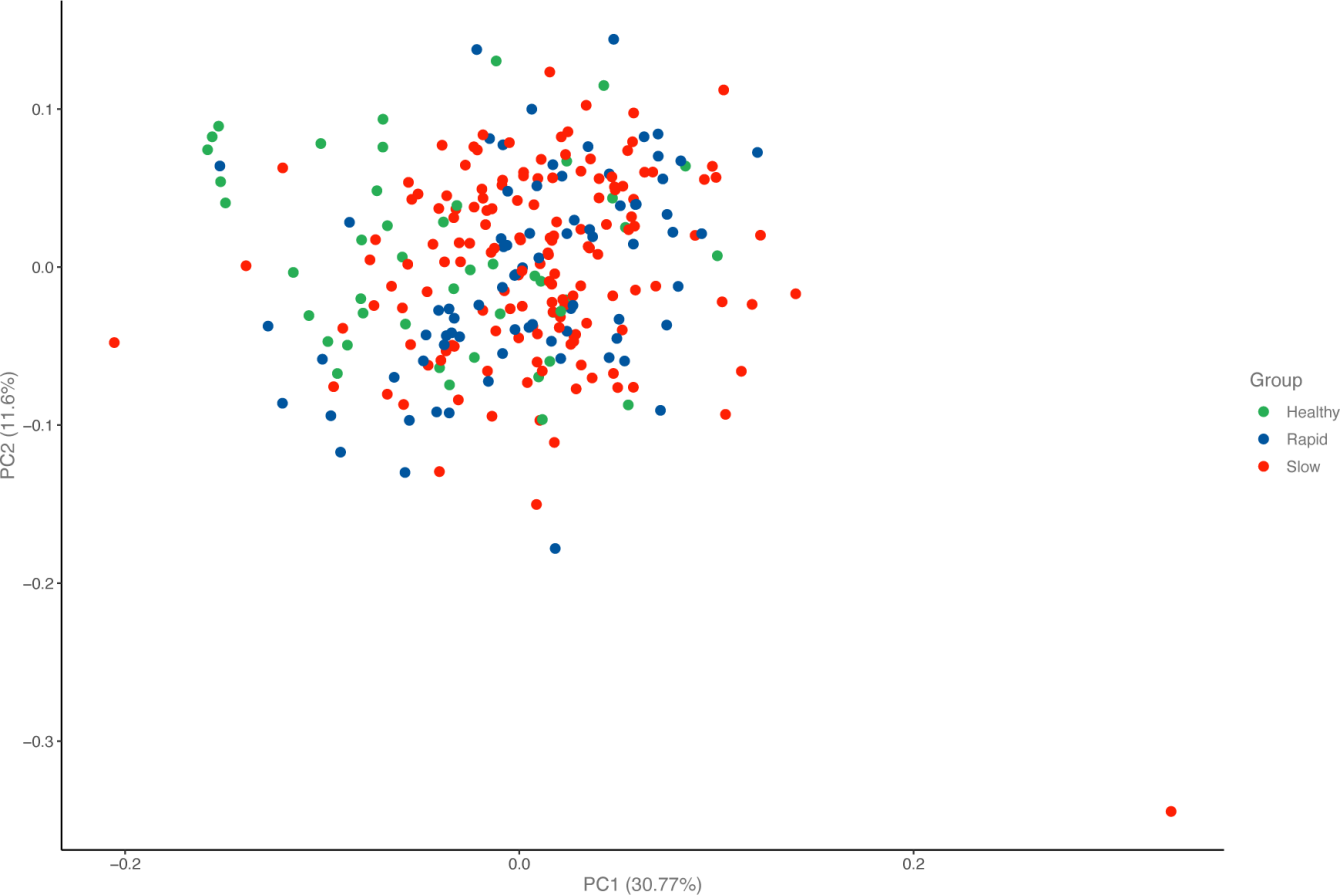
Principal component analysis samples in immune response panel.

**Figure 2:**
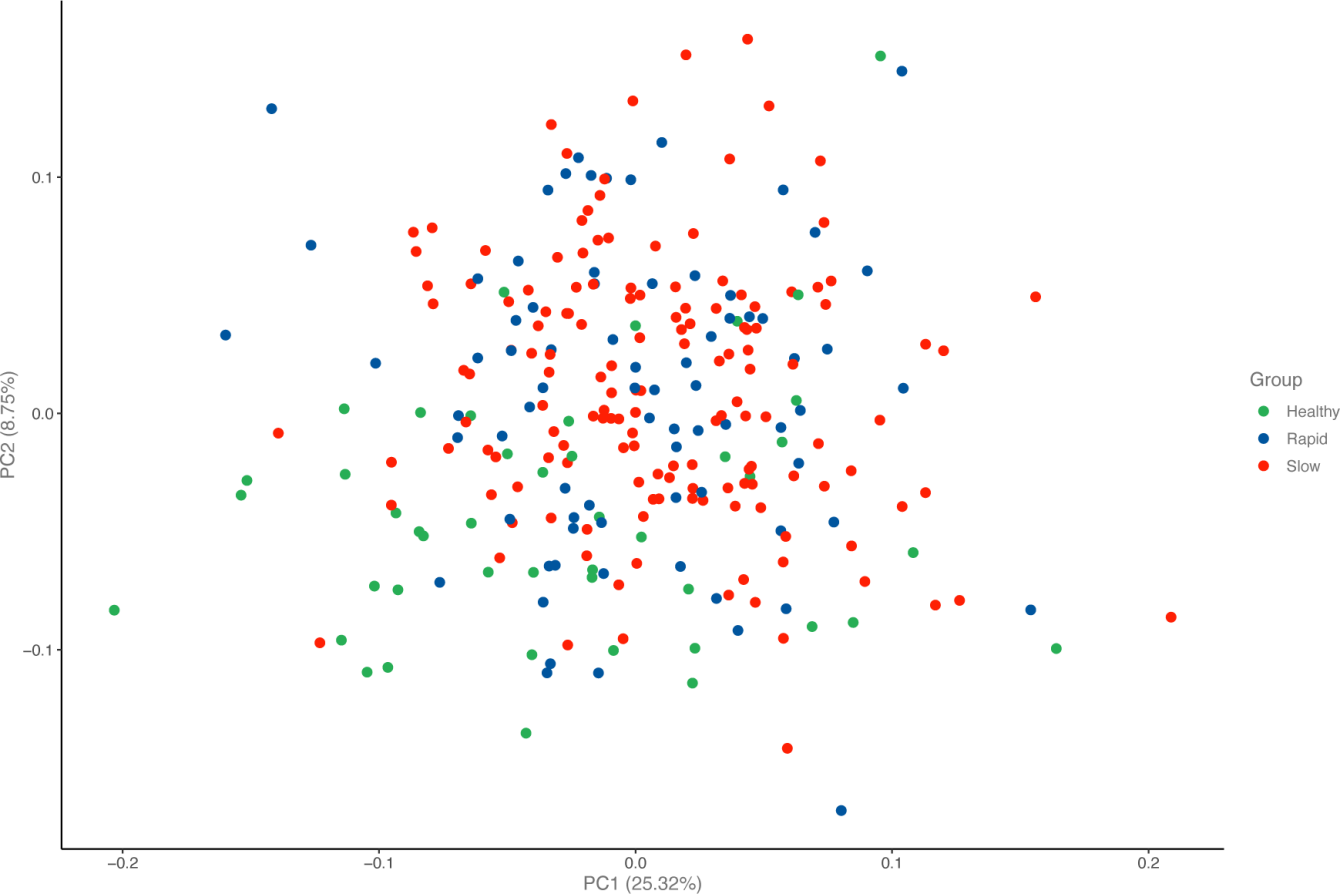
Principal component analysis samples in immune- oncology panel.

At baseline, 184 biomarkers were measured in the *Rapid*, *Slow*, and *Healthy* groups. The groups had no distinctive biomarker patterns that could separate them from each other (figures 1 and 2).

### Individual biomarkers

#### ANOVA: *Rapid* versus *Slow* versus *Healthy*

**Figure 3:**
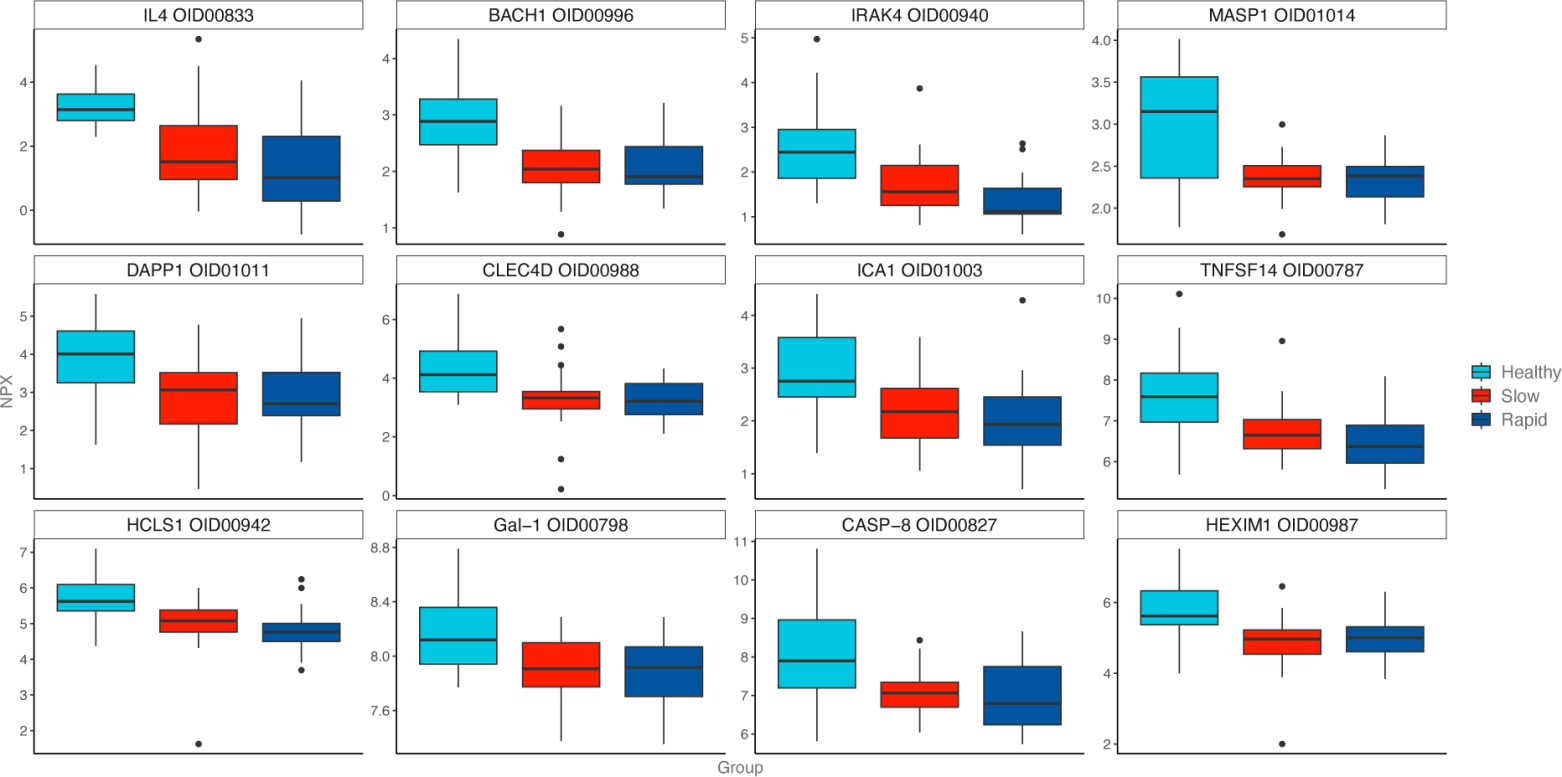
Boxplots of the 12 most significant biomarkers in ANOVA.

Sixty-four biomarkers differed in the ANOVA of *Rapid*, *Slow*, and *Healthy* (figure 3), with a similar outcome to the t-test findings. A post hoc analysis revealed higher levels of three proteins in group *Rapid* compared with *Slow*, including carbonic anhydrase 9 (CAIX, p<0.001), corticosteroid 11-beta-dehydrogenase isozyme 1 (HSD11B1, p<0.01), and tumor necrosis factor receptor superfamily member 21 (TNFRSF21, p<0.05) (see online supplemental table 5).

10.1136/bmjdrc-2023-003924.supp5 Supplementary data

#### Welch t-test: *T1D* cohort versus *Healthy* controls

**Figure 4:**
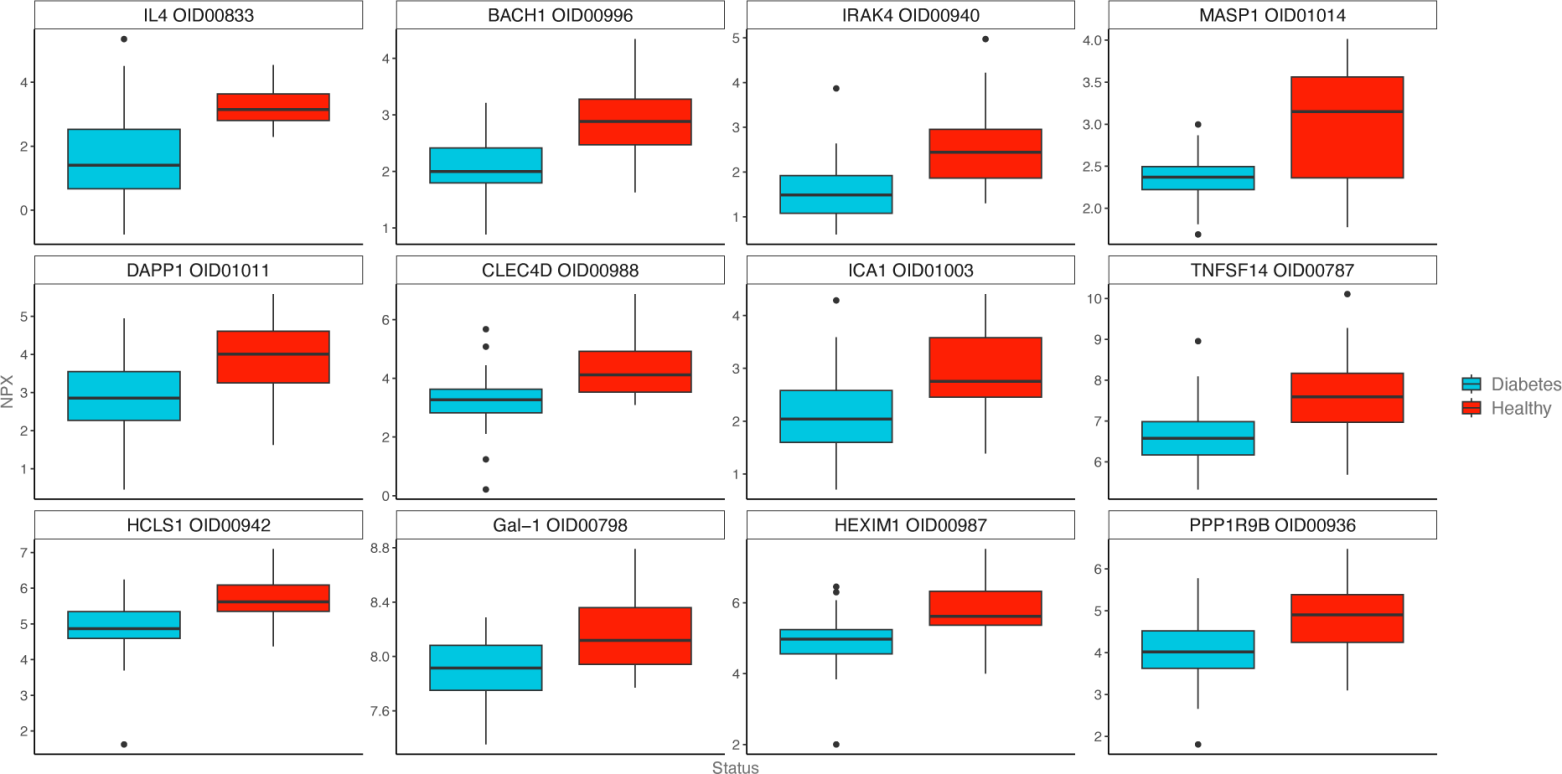
Boxplots of the 12 most significant biomarkers comparing groups *T1D* with *Healthy*. T1D, type 1 diabetes.

Eighty-one biomarkers differed significantly between *T1D* (*Rapid* and *Slow* as one group) and *Healthy* at baseline (online supplemental table 4). Eleven of the 81 significant biomarkers were higher, and 70 were lower in the *T1D* cohort compared with *Healthy*. Cytokines with notable associations to the pathogenesis or prevention of type 1 diabetes included C-type lectin domain family 4 member D (CLEC4D, p<0.0001),^13^ interleukin (IL)-4 (p<0.0001),^14^ IL-12^15^ (p<0.05), IL-13^16^ (p<0.05), galectin-1 (Gal-1, p<0.0001),^17^ tumor necrosis factor superfamily member 14 (TNFSF14, p<0.0001),^18^ islet cell antigen 1 (ICA1, p<0.0001),^19^ CD40 ligand^20^ (p<0.05), mannan-binding lectin-associated serine proteases (MASP-1, p<0.0001),^21^ peroxiredoxin-1 (PRDX1, p<0.01),^22^ and latency-associated protein transforming growth factor beta 1 (LAP-TGF-beta 1, p<0.001).^23^ (figure 4)

10.1136/bmjdrc-2023-003924.supp4 Supplementary data

#### Linear mixed effects model (LMER)

**Figure 5:**
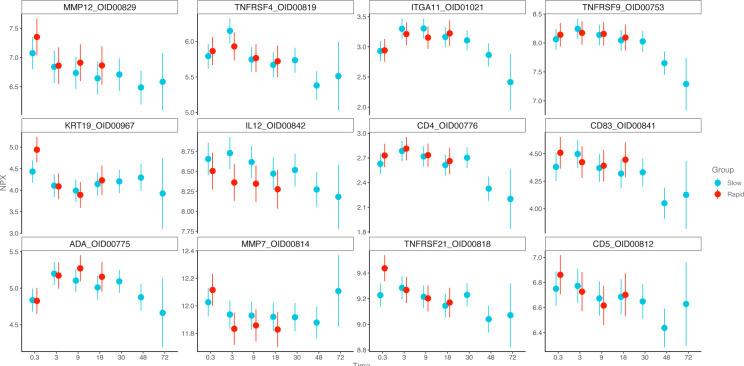
Point range plots of the top 12 most significant proteins for the Time term in linear mixed effects model.

Applying an LMER, the terms Time and Group (Rapid/Slow) were set as fixed and patient ID as random effects, respectively. Group term means that there is a difference between the groups independent of time, a significant Time term means that there is a difference over time independent of the group, and a significant interaction (Time:Group) effect means that change over time depends on the group. Twenty-five proteins differed for the *Time* term (figure 5), including previously identified biomarkers of interest, IL-12 from debut and 3 to 18 months (both p<0.05), and Gal-1 from debut to 3 months (p<0.001) and from 3 to 18 months (p<0.05), with no difference for the *Group* term. One protein, the coxsackievirus B–adenovirus receptor (CAR), increased for the *Time:Group* interaction term, in the group *Rapid* from diagnosis to 9 (p<0.001) and 18 months (p<0.01), respectively. Integrin beta-6 (ITGB6) also increased in the *Time:Group* term for group *Rapid* but was not statistically significant after adjusting for multiple testing (p=0.07).

## Conclusions/discussion

The rate of progression to complete beta cell failure in type 1 diabetes is heterogeneous. The variation is associated with clinical characteristics, but differences in the underlying biochemistry remain largely undefined. Identifying biomarkers associated with the disease progression rate could unveil underlying mechanisms and be of prognostic value, especially when selecting suitable intervention therapies. To investigate this, we analyzed 184 biomarkers by PEA in 46 children newly diagnosed with type 1 diabetes until loss of residual C-peptide secretion and compared baseline values with 45 healthy controls. As the study was performed retrospectively, we could classify participants with type 1 diabetes as progressing rapidly or slowly.

When comparing *T1D* with *Healthy*, 81 of the 184 analyzed proteins differed significantly. This notably includes several proteins associated with type 1 diabetes pathogenesis or protection and mainly confirms earlier findings. IL-4 was lower in the *T1D* cohort and has been associated with both protection against autoimmunity^14^ as well as type 1 diabetes debut,^24 25^ indeed, the IL-4 receptor is expressed in pancreatic islets.^26^ IL-13, which is mostly known as a Th2 cytokine with anti-inflammatory properties,^27^ was found to be higher in the *T1D* cohort, which was somewhat surprising considering that previous studies have found that the IL-13 production is decreased in patients with type 1 diabetes and individuals at risk of type 1 diabetes.^28 29^ Also, experimental in vitro studies have found that IL-13 can reduce beta-cell apoptosis.^30^ CLEC4D was lower in the *T1D* cohort, consistent with previous research associating low CLEC4D with proinflammatory states.^31^ This corroborates recent findings linking significantly lower concentrations of CLEC4D with positive type 1 diabetes autoantibodies in human pancreata.^13^ PRDX1^22^ is an antioxidant with immunoregulatory properties that were lower in *T1D*. LAP-TGF-beta 1^23^ is the upstream pro-protein of immunoregulator TGF-beta 1 and was lower in *T1D*. Notably, TGF-beta 1 dissociates from LAP and becomes active on interaction with primarily integrin ITGAV:ITGB6^32^ and can promote Th17 or Treg differentiation in a concentration-dependent manner. High concentrations of TGF-beta 1 are shown to downregulate IL-17 expression in favor of Tregs, while low concentrations can promote Th17 differentiation.^33^

Soluble lectin Gal-1 was lower in the *T1D* cohort and is an anti-inflammatory cytokine^34^ involved in several autoimmune diseases, including type 1 diabetes.^17^ In addition, studies in non-obese diabetic mice associate Gal-1^35^ and TNFSF14 (aka LIGHT)^18^ with reversal of beta cell autoimmunity and insulitis, respectively.

Overall, lower levels of IL-4, Gal-1, TNFSF14, and higher levels of IL-12 in the *T1D* cohort are in line with a Th1-dominated immune response and previously published data. Higher IL-13 in the *T1D* cohort, a cytokine primarily associated with Th2 cells and immune tolerance, was unexpected and seemingly at odds with the Th1/Th2 paradigm of autoimmunity.

Our results primarily point to a lack of protective proteins in the *T1D* cohort compared with *Healthy*. In fact, cytokine levels were mostly lower in cohort *T1D*, possibly representing an inability to moderate an overzealous immune system attacking pancreatic beta cells. Incongruously, other biomarkers associated with type 1 diabetes pathogenesis, such as autoantibody ICA1,^19^ immune cell activator CD40-ligand,^20^ and complement activator MASP-1,^21^ that can be raised in type 1 diabetes, were also lower in the *T1D* group, illustrating the complexity and heterogeneity of the type 1 diabetes pathogenesis.

The ANOVA showed higher levels of proteins CAIX, HSD11B1, and TNFRSF21 in group *Rapid* versus *Slow*. CAIX is involved in the L-Arginine/Nitric Oxide pathway and has previously been found to be altered in children with type 1 diabetes,^36^ but it has not previously been associated with rapid disease progression per se. HSD11B1 is a catalyst for the reversible conversion of cortisone to cortisol and is expressed in various tissues. Nocturnal HSD11B1 has previously been found to be higher in children with type 1 diabetes^37^ and has been found to increase insulin resistance.^38^ HSD11B1 is also expressed in alpha and beta cells and is demonstrated to blunt glucose-stimulated insulin secretion.^39 40^ Hence, a higher HSD11B1 could speed up beta cell exhaustion via increased peripheral insulin resistance and reduced insulin secretion. This may partly explain the lower stimulated C-peptide in the *Rapid* group, suggesting that there could be a functional impairment of beta cells in addition to direct destruction. TNFRSF21 is involved with the negative regulation of Th2-cell activation and cytokine release.^41^ Upregulated TNFRSF21 and lower activation of the immune modulatory Th2-dependent pathway are in line with a more aggressive autoimmune attack in group *Rapid*.

In the linear mixed model, we found that none of the analyzed proteins differed in the *Group* term (*Rapid* vs *Slow*). However, 25 proteins, including ITGB6, IL-12, and Gal-1, differed in the *Time* term, suggesting an involvement in disease progression. Interestingly, ITGB6 has recently been found to be a target of autoantibodies in inflammatory bowel disease^42^ suggesting that it could be involved in autoimmunity.

Interestingly, in the combined *Time:Group* interaction term only one marker, namely CAR, was altered significantly and found to increase in the *Rapid* group from debut to 9 and 18 months, respectively. ITGB6 was also altered over time depending on the group, but this was not significant after adjusting for multiple testing. It is, however, striking that CAR and ITGB6 are functionally similar, being trans-membrane, ligand-binding, signaling proteins involved in cell–cell interaction and autoimmunity. CARs are present on beta cells’ surface and are a known port of entry for adenovirus and coxsackievirus, potentially triggering autoimmunity and insulitis.^43 44^ ITGB6 is part of a dimer receptor together with integrin alpha 5 (ITGAV) that binds coxsackievirus and is expressed in pancreatic islet cells.^45^ It is striking that this port of entry for viruses also activates TGF-beta 1, a known regulator of the T-helper 17 cell (Th17) pathway. Numerous epidemiological and clinical investigations support an association between enteroviruses, particularly coxsackievirus B, and autoimmune type 1 diabetes.^46 47^ Although the mechanism remains incompletely understood, experimental findings are suggestive of molecular mimicry or bystander T-cell activation.^47^ In this study, the change in CARs is specific to patients with rapidly progressing type 1 diabetes, which suggests an association between CARs and disease progression.

A strength of this study is the collection of initial blood samples shortly after type 1 diabetes diagnosis, unlike the wider definition of new-onset type 1 diabetes, which in some studies may extend up to a year after diagnosis. A limitation of the study is the inherent difficulty in interpreting how cytokine levels in peripheral blood mirror the immune response local to the pancreas. However, this approach does not compromise our aim to find prognostic biomarkers of type 1 diabetes progression rate. Furthermore, it is a limitation that 12 of 184 proteins (eg, IL-35, interferon, tumor necrosis factor) failed QC of the normalization between assays and were excluded from analyses. However, these biomarkers are deemed unlikely to separate the overlapping PCA plots. Also, the sample size of the study could limit the detection of more discrete differences between the groups.

In summary, the main aim of our study was to find biomarkers predictive of C-peptide decline in children with new-onset type 1 diabetes, but of the analyzed biomarkers, we found no clear separation in patterns that could separate the *Rapid*, *Slow*, and *Healthy* groups at baseline. However, we did find some markers which may be useful in predicting the decline of C-peptide. Also, these could potentially be important for our understanding of the type 1 diabetes pathogeneses, which merits further investigation.

## Data Availability

Data are available upon reasonable request.
