# Supplementary material for: Predictive biomarkers of rapidly developing insulin deficiency in children with type 1 diabetes
Source: BMJ Open Diabetes Res Care. 2024 Feb 27;12(1):e003924. doi: 10.1136/bmjdrc-2023-003924 (PMC10900379; doi:10.1136/bmjdrc-2023-003924)
Supplement: Supplementary data [file bmjdrc-2023-003924supp001.pdf]

Protein assay list

Olink® Target 96 Immuno-Oncology

Product number: 95311

Adenosine deaminase (ADA)	P00813	C-X-C motif chemokine 11 (CXCL11)	O14625
Adhesion G-protein coupled receptor G1 (ADGRG1)	Q9Y653	C-X-C motif chemokine 13 (CXCL13)	O43927
Angiopoietin-1 (ANGPT1)	Q15389	C-X-C motif chemokine 5 (CXCL5)	P42830
Angiopoietin-1 receptor (TIE2)	Q02763	C-X-C motif chemokine 9 (CXCL9)	Q07325
Angiopoietin-2 (ANGPT2)	O15123	Cytotoxic and regulatory T-cell molecule (CRTAM)	O95727
Arginase-1 (ARG1)	P05089	Decorin (DCN)	P07585
Carbonic anhydrase 9 (CAIX)	Q16790	Fibroblast growth factor 2 (FGF2)	P09038
Caspase-8 (CASP-8)	Q14790	Fractalkine (CX3CL1)	P78423
C-C motif chemokine 13 (MCP-4)	Q99616	Galectin-1 (Gal-1)	P09382
C-C motif chemokine 17 (CCL17)	Q92583	Galectin-9 (Gal-9)	O00182
C-C motif chemokine 19 (CCL19)	Q99731	Granzyme A (GZMA)	P12544
C-C motif chemokine 2 (MCP-1)	P13500	Granzyme B (GZMB)	P10144
C-C motif chemokine 20 (CCL20)	P78556	Granzyme H (GZMH)	P20718
C-C motif chemokine 23 (CCL23)	P55773	Heme oxygenase 1 (HO-1)	P09601
C-C motif chemokine 3 (CCL3)	P10147	Hepatocyte growth factor (HGF)	P14210
C-C motif chemokine 4 (CCL4)	P13236	ICOS ligand (ICOSLG)	O75144
C-C motif chemokine 7 (MCP-3)	P80098	Interferon gamma (IFN-gamma)	P01579
C-C motif chemokine 8 (MCP-2)	P80075	Interleukin-1 alpha (IL-1 alpha)	P01583
CD27 antigen (CD27)	P26842	Interleukin-10 (IL10)	P22301
CD40 ligand (CD40-L)	P29965	Interleukin-12 (IL12)	P29459, P29460
CD40L receptor (CD40)	P25942	Interleukin-12 receptor subunit beta-1 (IL12RB1)	P42701
CD70 antigen (CD70)	P32970	Interleukin-13 (IL13)	P35225
CD83 antigen (CD83)	Q01151	Interleukin-15 (IL15)	P40933
C-X-C motif chemokine 1 (CXCL1)	P09341	Interleukin-18 (IL18)	Q14116
C-X-C motif chemokine 10 (CXCL10)	P02778	Interleukin-2 (IL2)	P60568

Table continues on reverse ▶

Interleukin-33 (IL33)	O95760	Pleiotrophin (PTN)	P21246
Interleukin-4 (IL4)	P05112	Pro-epidermal growth factor (EGF)	P01133
Interleukin-5 (IL5)	P05113	Programmed cell death 1 ligand 1 (PD-L1)	Q9NZQ7
Interleukin-6 (IL6)	P05231	Programmed cell death 1 ligand 2 (PD-L2)	Q9BQ51
Interleukin-7 (IL7)	P13232	Programmed cell death protein 1 (PDCD1)	Q15116
Interleukin-8 (IL8)	P10145	Stromal cell-derived factor 1 (CXCL12)	P48061
Killer cell immunoglobulin-like receptor 3DL1 (KIR3DL1)	P43629	T-cell surface glycoprotein CD4 (CD4)	P01730
Latency-associated peptide transforming growth factor beta-1 (LAP TGF-beta-1)	P01137	T-cell surface glycoprotein CD5 (CD5)	P06127
Lymphocyte activation gene 3 protein (LAG3)	P18627	T-cell surface glycoprotein CD8 alpha chain (CD8A)	P01732
Lysosome-associated membrane glycoprotein 3 (LAMP3)	Q9UQV4	T-cell-specific surface glycoprotein CD28 (CD28)	P10747
Macrophage colony-stimulating factor 1 (CSF-1)	P09603	TNF-related apoptosis-inducing ligand (TRAIL)	P50591
Macrophage metalloproteinase-12 (MMP12)	P39900	Tumor necrosis factor (TNF)	P01375
Matrix metalloproteinase-7 (MMP7)	P09237	Tumor necrosis factor ligand superfamily member 12 (TWEAK)	O43508
MHC class I polypeptide-related sequence A/B (MIC-A/B)	Q29983, Q29980	Tumor necrosis factor ligand superfamily member 14 (TNFSF14)	O43557
Mucin-16 (MUC-16)	Q8WXI7	Tumor necrosis factor ligand superfamily member 6 (FASLG)	P48023
Natural cytotoxicity triggering receptor (NCR1)	O76036	Tumor necrosis factor receptor superfamily member 12A (TNFRSF12A)	Q9NP84
Natural killer cell receptor 2B4 (CD244)	Q9BZW8	Tumor necrosis factor receptor superfamily member 21 (TNFRSF21)	O75509
Natural killer cells antigen CD94 (KLRD1)	Q13241	Tumor necrosis factor receptor superfamily member 4 (TNFRSF4)	P43489
Nitric oxide synthase, endothelial (NOS3)	P29474	Tumor necrosis factor receptor superfamily member 9 (TNFRSF9)	Q07011
Placenta growth factor (PGF)	P49763	Vascular endothelial growth factor A (VEGFA)	P15692
Platelet-derived growth factor subunit B (PDGF subunit B)	P01127	Vascular endothelial growth factor receptor 2 (VEGFR-2)	P35968

For more details visit www.olink.com/immuno-onc

www.olink.com

For research use only. Not for use in diagnostic procedures.
This product includes a license for non-commercial use. Commercial users may require additional licenses. Please contact Olink Proteomics AB for details.
There are no warranties, expressed or implied, which extend beyond this description. Olink Proteomics AB is not liable for property damage, personal injury, or economic loss caused by this product.
Olink® is a registered trademark of Olink Proteomics AB.

© 2017–2022 Olink Proteomics AB. All third party trademarks are the property of their respective owners.

Olink Proteomics, Dag Hammarskjölds väg 52B , SE-752 37 Uppsala, Sweden
1047, v2.0, 2022-06-14
