# Supplementary material for: Predictive biomarkers of rapidly developing insulin deficiency in children with type 1 diabetes
Source: BMJ Open Diabetes Res Care. 2024 Feb 27;12(1):e003924. doi: 10.1136/bmjdrc-2023-003924 (PMC10900379; doi:10.1136/bmjdrc-2023-003924)
Supplement: Supplementary data [file bmjdrc-2023-003924supp002.pdf]

Olink®

Protein assay list

Olink® Target 96 Immune Response

Product number: 95320

Allergin-1 (MILR1)	Q7Z6M3	Eotaxin (CCL11)	P51671
Amphiregulin (AR) (AREG)	P15514	Eukaryotic translation initiation factor 4 gamma 1 (EIF4G1)	Q04637
Aryl hydrocarbon receptor nuclear translocator (ARNT)	P27540	Eukaryotic translation initiation factor 5A-1 (EIF5A)	P63241
Baculoviral IAP repeat-containing protein 2 (BIRC2)	Q13490	Fc receptor-like protein 3 (FCRL3)	Q96P31
Beta-galactosidase (GLB1)	P16278	Fc receptor-like protein 6 (FCRL6)	Q6DN72
Butyrophilin subfamily 3 member A2 (BTN3A2)	P78410	Fibroblast growth factor 2 (FGF2)	P09038
CD83 antigen (CD83)	Q01151	FXYD domain-containing ion transport regulator 5 (FXYD5)	Q96DB9
Contactin-associated protein-like 2 (CNTNAP2)	Q9UHC6	Hematopoietic lineage cell-specific protein (HCLS1)	P14317
Corneodesmosin (CDSN)	Q15517	Histamine N-methyltransferase (HNMT)	P50135
Corticosteroid 11-beta-dehydrogenase isozyme 1 (HSD11B1)	P28845	Importin subunit alpha-5 (KPNA1)	P52294
Coxsackievirus and adenovirus receptor (CXADR)	P78310	Inactive dipeptidyl peptidase 10 (DPP10)	Q8N608
C-type lectin domain family 4 member A (CLEC4A)	Q9UMR7	Integral membrane protein 2A (ITM2A)	O43736
C-type lectin domain family 4 member C (CLEC4C)	Q8WTT0	Integrin alpha-6 (ITGA6)	P23229
C-type lectin domain family 4 member D (CLEC4D)	Q8WXI8	Integrin alpha-11 (ITGA11)	Q9UKX5
C-type lectin domain family 4 member G (CLEC4G)	Q6UXB4	Integrin beta-6 (ITGB6)	P18564
C-type lectin domain family 6 member A (CLEC6A)	Q6EIG7	Interferon lambda receptor 1 (IFNLR1)	Q8IU57
C-type lectin domain family 7 member A (CLEC7A)	Q9BXN2	Interferon regulatory factor 9 (IRF9)	Q00978
Cytoskeleton-associated protein 4 (CKAP4)	Q07065	Interleukin-1 receptor-associated kinase 1 (IRAK1)	P51617
Diacylglycerol kinase zeta (DGKZ)	Q13574	Interleukin-1 receptor-associated kinase 4 (IRAK4)	Q9NWZ3
Discoidin, CUB and LCCL domain-containing protein 2 (DCBLD2)	Q96PD2	Interleukin-5 (IL5)	P05113
DNA fragmentation factor subunit alpha (DFFA)	O00273	Interleukin-6 (IL6)	P05231
Dual adapter for phosphotyrosine and 3-phosphotyrosine and 3-phosphoinositide (DAPP1)	Q9UN19	Interleukin-10 (IL10)	P22301
Dynactin subunit 1 (DCTN1)	Q14203	Interleukin-12 receptor subunit beta-1 (IL12RB1)	P42701
E3 ubiquitin-protein ligase TRIM21 (TRIM21)	P19474	Islet cell autoantigen 1 (ICA1)	Q05084
Egl nine homolog 1 (EGLN1)	Q9GZT9	Keratin, type I cytoskeletal 19 (KRT19)	P08727

Table continues on reverse ▶

Leukocyte immunoglobulin-like receptor subfamily B member 4 (LILRB4)	Q8NHJ6	Protein HEXIM1 (HEXIM1)	O94992
Lymphocyte activation gene 3 protein (LAG3)	P18627	Protein kinase C theta type (PRKCQ)	Q04759
Lymphocyte antigen 75 (LY75)	O60449	Protein sprouty homolog 2 (SPRY2)	O43597
Lysosome-associated membrane glycoprotein 3 (LAMP3)	Q9UQV4	Protein-arginine deiminase type-2 (PADI2)	Q9Y2J8
Mannan-binding lectin serine protease 1 (MASP1)	P48740	SH2 domain-containing protein 1A (SH2D1A)	O60880
Merlin (NF2)	P35240	SH2B adapter protein 3 (SH2B3)	Q9UQQ2
Methylated-DNA--protein-cysteine methyltransferase (MGMT)	P16455	Signaling threshold-regulating transmembrane adapter 1 (SIT1)	Q9Y3P8
Natural cytotoxicity triggering receptor 1 (NCR1)	O76036	SRSF protein kinase 2 (SRPK2)	P78362
Natural killer cells antigen CD94 (KLRD1)	Q13241	Stanniocalcin-1 (STC1)	P52823
Neurabin-2 (PPP1R9B)	Q96SB3	Stromal cell-derived factor 1 (CXCL12)	P48061
Neurotrophin-4 (NTF4)	P34130	T-cell-specific surface glycoprotein CD28 (CD28)	P10747
Nuclear factor of activated T-cells, cytoplasmic 3 (NFATC3)	Q12968	Thioredoxin-dependent peroxide reductase, mitochondrial (PRDX3)	P30048
Parathyroid hormone/parathyroid hormone-related peptide receptor (PTH1R)	Q03431	TNF receptor-associated factor 2 (TRAF2)	Q12933
PC4 and SFRS1-interacting protein (PSIP1)	O75475	TRAF family member-associated NF-kappa-B activator (TANK)	Q92844
Peroxiredoxin-1 (PRDX1)	Q06830	Transcription factor AP-1 (JUN)	P05412
Peroxiredoxin-5, mitochondrial (PRDX5)	P30044	Transcription regulator protein BACH1 (BACH1)	O14867
Phosphoinositide 3-kinase adapter protein 1 (PIK3AP1)	Q6ZUJ8	Triggering receptor expressed on myeloid cells 1 (TREM1)	Q9NP99
Plexin-A4 (PLXNA4)	Q9HCM2	Tripartite motif-containing protein 5 (TRIM5)	Q9C035
Polypeptide N-acetylgalactosaminyltransferase 3 (GALNT3)	Q14435	Tryptase alpha/beta-1 (TPSAB1)	Q15661
Probable ATP-dependent RNA helicase DDX58 (DDX58)	O95786	Tumor necrosis factor receptor superfamily member EDAR (EDAR)	Q9UNE0
Protein FAM3B (FAM3B)	P58499	Zinc finger and BTB domain-containing protein 16 (ZBTB16)	Q05516

For more details visit www.olink.com/immune-response

www.olink.com

For research use only. Not for use in diagnostic procedures.
This product includes a license for non-commercial use. Commercial users may require additional licenses. Please contact Olink Proteomics AB for details.
There are no warranties, expressed or implied, which extend beyond this description. Olink Proteomics AB is not liable for property damage, personal injury, or economic loss caused by this product.
Olink® is a registered trademark of Olink Proteomics AB.

© 2017–2022 Olink Proteomics AB. All third party trademarks are the property of their respective owners.
Olink Proteomics, Dag Hammarskjölds väg 52B , SE-752 37 Uppsala, Sweden
1051, v2.0, 2022-06-14
