# Supplementary material for: Predictive biomarkers of rapidly developing insulin deficiency in children with type 1 diabetes
Source: BMJ Open Diabetes Res Care. 2024 Feb 27;12(1):e003924. doi: 10.1136/bmjdrc-2023-003924 (PMC10900379; doi:10.1136/bmjdrc-2023-003924)
Supplement: Supplementary data [file bmjdrc-2023-003924supp003.pdf]

Analysis

Only proteins that were normalized were used in the following analysis. In Table 2, assays that were not included in the analysis are listed.

Table 2: The lists of unnormalized proteins were not included in the analysis.

Assay	OlinkID	Panel
IFN-beta	OID00774	Olink Immuno-Oncology
IFN-gamma	OID00825	Olink Immuno-Oncology
IFN-gamma	OID05552	Olink Immuno-Oncology
IL-21	OID00834	Olink Immuno-Oncology
IL-35	OID00797	Olink Immuno-Oncology
IL15	OID05551	Olink Immuno-Oncology
KIR3DL1	OID05550	Olink Immuno-Oncology
LAG3	OID05553	Olink Immuno-Oncology
MUC-16	OID05549	Olink Immuno-Oncology
TNF	OID00838	Olink Immuno-Oncology
TNF	OID05554	Olink Immuno-Oncology
VEGFC	OID00784	Olink Immuno-Oncology
