# Supplementary material for: Predictive biomarkers of rapidly developing insulin deficiency in children with type 1 diabetes
Source: BMJ Open Diabetes Res Care. 2024 Feb 27;12(1):e003924. doi: 10.1136/bmjdrc-2023-003924 (PMC10900379; doi:10.1136/bmjdrc-2023-003924)
Supplement: Supplementary data [file bmjdrc-2023-003924supp005.pdf]

Table 5. ANOVA Rapid, Slow and Healthy adjusted for multiplicity.

Assay	statistic	p.value	Adj.P-value	Threshold
IL4	24,65	4,14E-09	7,38E-07	Sign
BACH1	21,16	3,14E-08	1,40E-06	Sign
IRAK4	21,24	2,96E-08	1,40E-06	Sign
MASP1	21,25	2,96E-08	1,40E-06	Sign
DAPP1	15,69	1,49E-06	5,30E-05	Sign
CLEC4D	15,19	2,16E-06	5,49E-05	Sign
ICA1	15,23	2,10E-06	5,49E-05	Sign
TNFSF14	14,75	3,05E-06	6,79E-05	Sign
HCLS1	13,47	7,90E-06	1,56E-04	Sign
Gal-1	12,14	2,24E-05	3,98E-04	Sign
CASP-8	11,83	2,85E-05	4,45E-04	Sign
HEXIM1	11,75	3,00E-05	4,45E-04	Sign
PPP1R9B	11,48	3,71E-05	5,08E-04	Sign
IL8	11,04	5,34E-05	6,18E-04	Sign
PIK3AP1	11,07	5,13E-05	6,18E-04	Sign
TRIM21	10,97	5,56E-05	6,18E-04	Sign
PRDX5	10,32	9,42E-05	9,86E-04	Sign
HSD11B1	9,88	1,34E-04	1,33E-03	Sign
CXCL12	9,65	1,63E-04	1,53E-03	Sign
GZMH	9,52	1,82E-04	1,62E-03	Sign
DCN	9,37	2,07E-04	1,67E-03	Sign
LAP TGF-beta-1	9,39	2,03E-04	1,67E-03	Sign
CLEC6A	9,00	2,77E-04	2,14E-03	Sign
CCL23	8,81	3,28E-04	2,43E-03	Sign
PSIP1	8,36	4,75E-04	3,38E-03	Sign
GLB1	8,16	5,60E-04	3,83E-03	Sign
DFFA	7,89	7,03E-04	4,47E-03	Sign
TNFRSF21	7,93	6,84E-04	4,47E-03	Sign
EGLN1	7,73	8,09E-04	4,64E-03	Sign
IRAK1	7,79	7,66E-04	4,64E-03	Sign
PRDX1	7,75	7,92E-04	4,64E-03	Sign
ANG-1	7,70	8,36E-04	4,65E-03	Sign
SIT1	7,41	1,06E-03	5,70E-03	Sign
HGF	7,26	1,22E-03	6,37E-03	Sign
TREM1	7,00	1,51E-03	7,69E-03	Sign
SRPK2	6,95	1,58E-03	7,81E-03	Sign
CAIX	6,89	1,67E-03	7,82E-03	Sign
KRT19	6,91	1,64E-03	7,82E-03	Sign
SPRY2	6,64	2,06E-03	9,41E-03	Sign
CLEC4A	6,54	2,25E-03	1,00E-02	Sign
EDAR	6,43	2,48E-03	1,05E-02	Sign
SH2D1A	6,44	2,45E-03	1,05E-02	Sign
ARG1	6,32	2,73E-03	1,13E-02	Sign

ADA	6,27	2,87E-03	1,16E-02	Sign
TRIM5	6,24	2,93E-03	1,16E-02	Sign
TWEAK	6,06	3,42E-03	1,32E-02	Sign
ITGA6	5,84	4,15E-03	1,57E-02	Sign
CD4	5,73	4,61E-03	1,71E-02	Sign
IRF9	5,66	4,88E-03	1,77E-02	Sign
MMP12	5,56	5,34E-03	1,90E-02	Sign
EIF4G1	5,14	7,72E-03	2,69E-02	Sign
BTN3A2	5,04	8,44E-03	2,89E-02	Sign
PRKCQ	5,02	8,65E-03	2,90E-02	Sign
BIRC2	4,84	1,02E-02	3,36E-02	Sign
DCTN1	4,79	1,06E-02	3,42E-02	Sign
ITM2A	4,65	1,21E-02	3,84E-02	Sign
NCR1	4,61	1,24E-02	3,88E-02	Sign
FXYD5	4,59	1,27E-02	3,89E-02	Sign
CXCL13	4,51	1,37E-02	4,15E-02	Sign
ICOSLG	4,41	1,50E-02	4,45E-02	Sign
CKAP4	4,36	1,56E-02	4,55E-02	Sign
GZMB	4,27	1,70E-02	4,89E-02	Sign
DDX58	4,23	1,76E-02	4,93E-02	Sign
VEGFA	4,23	1,77E-02	4,93E-02	Sign
IL12	4,15	1,90E-02	5,12E-02	NS
MIC-A/B	4,16	1,87E-02	5,12E-02	NS
CCL4	4,07	2,04E-02	5,36E-02	NS
CD40-L	4,07	2,05E-02	5,36E-02	NS
CLEC4G	4,03	2,11E-02	5,44E-02	NS
CD83	3,99	2,20E-02	5,61E-02	NS
IL13	3,90	2,38E-02	5,97E-02	NS
EGF	3,87	2,46E-02	6,08E-02	NS
FGF2	3,84	2,52E-02	6,16E-02	NS
IL7	3,75	2,74E-02	6,24E-02	NS
LY75	3,76	2,70E-02	6,24E-02	NS
PDGF subunit B	3,77	2,69E-02	6,24E-02	NS
TANK	3,76	2,71E-02	6,24E-02	NS
ZBTB16	3,78	2,66E-02	6,24E-02	NS
IL12RB1	3,73	2,80E-02	6,31E-02	NS
IL-1 alpha	3,70	2,86E-02	6,36E-02	NS
CCL3	3,64	3,04E-02	6,69E-02	NS
KPNA1	3,53	3,34E-02	7,24E-02	NS
CCL19	3,36	3,94E-02	8,45E-02	NS
IL10	3,25	4,33E-02	9,18E-02	NS
CX3CL1	3,17	4,68E-02	9,80E-02	NS
TIE2	3,16	4,73E-02	9,80E-02	NS
CCL20	3,12	4,94E-02	1,01E-01	NS
DCBLD2	3,08	5,08E-02	1,03E-01	NS
ADGRG1	2,97	5,65E-02	1,13E-01	NS

NF2	2,94	5,82E-02	1,15E-01	NS
ITGB6	2,90	6,00E-02	1,17E-01	NS
CLEC7A	2,87	6,20E-02	1,20E-01	NS
AREG	2,85	6,29E-02	1,20E-01	NS
ANGPT2	2,77	6,79E-02	1,29E-01	NS
VEGFR-2	2,64	7,74E-02	1,45E-01	NS
NCR1	2,60	8,03E-02	1,49E-01	NS
IL6	2,58	8,18E-02	1,50E-01	NS
HO-1	2,54	8,43E-02	1,53E-01	NS
CDSN	2,53	8,54E-02	1,54E-01	NS
LAMP3	2,49	8,88E-02	1,58E-01	NS
MGMT	2,41	9,53E-02	1,68E-01	NS
GALNT3	2,36	1,01E-01	1,74E-01	NS
IL33	2,37	9,99E-02	1,74E-01	NS
IL6	2,33	1,04E-01	1,77E-01	NS
CCL11	2,30	1,06E-01	1,80E-01	NS
HNMT	2,11	1,27E-01	2,12E-01	NS
LILRB4	2,11	1,27E-01	2,12E-01	NS
CXCL5	2,07	1,33E-01	2,17E-01	NS
FGF2	2,07	1,32E-01	2,17E-01	NS
EIF5A	2,06	1,34E-01	2,17E-01	NS
FAM3B	1,99	1,43E-01	2,29E-01	NS
IFNLR1	1,95	1,48E-01	2,35E-01	NS
SH2B3	1,92	1,53E-01	2,40E-01	NS
GZMA	1,90	1,55E-01	2,42E-01	NS
FCRL3	1,76	1,79E-01	2,77E-01	NS
CXCL1	1,71	1,88E-01	2,88E-01	NS
ARNT	1,62	2,03E-01	3,09E-01	NS
PLXNA4	1,60	2,08E-01	3,14E-01	NS
PDCD1	1,59	2,10E-01	3,14E-01	NS
FASLG	1,54	2,21E-01	3,26E-01	NS
IL18	1,53	2,21E-01	3,26E-01	NS
CD5	1,40	2,51E-01	3,66E-01	NS
CCL17	1,32	2,72E-01	3,94E-01	NS
PTN	1,31	2,76E-01	3,96E-01	NS
CD8A	1,27	2,85E-01	4,06E-01	NS
CNTNAP2	1,21	3,03E-01	4,28E-01	NS
CXCL12	1,18	3,11E-01	4,33E-01	NS
MCP-4	1,18	3,11E-01	4,33E-01	NS
PD-L1	1,15	3,21E-01	4,43E-01	NS
MCP-1	1,13	3,27E-01	4,48E-01	NS
IL10	1,12	3,31E-01	4,50E-01	NS
LAG3	1,09	3,42E-01	4,61E-01	NS
MILR1	1,04	3,58E-01	4,75E-01	NS
TNFRSF12A	1,05	3,55E-01	4,75E-01	NS
TRAF2	1,02	3,64E-01	4,79E-01	NS

CD27	1,01	3,68E-01	4,82E-01	NS
FCRL6	0,93	3,98E-01	5,17E-01	NS
IL2	0,89	4,16E-01	5,29E-01	NS
KLRD1	0,89	4,16E-01	5,29E-01	NS
TPSAB1	0,90	4,11E-01	5,29E-01	NS
NFATC3	0,83	4,39E-01	5,54E-01	NS
CSF-1	0,82	4,46E-01	5,59E-01	NS
CD83	0,78	4,60E-01	5,73E-01	NS
CLEC4C	0,76	4,69E-01	5,75E-01	NS
PRDX3	0,77	4,68E-01	5,75E-01	NS
CD70	0,76	4,73E-01	5,76E-01	NS
STC1	0,68	5,10E-01	6,18E-01	NS
IL12RB1	0,66	5,21E-01	6,23E-01	NS
LAMP3	0,66	5,21E-01	6,23E-01	NS
TNFRSF4	0,65	5,25E-01	6,23E-01	NS
TNFRSF9	0,61	5,46E-01	6,43E-01	NS
PD-L2	0,60	5,51E-01	6,45E-01	NS
CD244	0,58	5,65E-01	6,49E-01	NS
NOS3	0,58	5,62E-01	6,49E-01	NS
PGF	0,58	5,63E-01	6,49E-01	NS
CXCL11	0,52	5,99E-01	6,84E-01	NS
JUN	0,48	6,18E-01	7,01E-01	NS
Gal-9	0,44	6,45E-01	7,26E-01	NS
DGKZ	0,38	6,82E-01	7,63E-01	NS
DPP10	0,37	6,92E-01	7,69E-01	NS
MMP7	0,36	6,97E-01	7,70E-01	NS
MCP-2	0,35	7,04E-01	7,73E-01	NS
PADI2	0,33	7,20E-01	7,86E-01	NS
KLRD1	0,32	7,24E-01	7,86E-01	NS
CXADR	0,23	7,96E-01	8,58E-01	NS
IL5	0,22	8,00E-01	8,58E-01	NS
CXCL10	0,22	8,06E-01	8,59E-01	NS
NTF4	0,20	8,18E-01	8,67E-01	NS
CD40	0,19	8,23E-01	8,67E-01	NS
PTH1R	0,16	8,52E-01	8,92E-01	NS
CD28	0,13	8,81E-01	9,10E-01	NS
CRTAM	0,12	8,87E-01	9,10E-01	NS
ITGA11	0,12	8,87E-01	9,10E-01	NS
TRAIL	0,12	8,89E-01	9,10E-01	NS
CD28	0,07	9,29E-01	9,44E-01	NS
CXCL9	0,05	9,49E-01	9,60E-01	NS
IL5	0,04	9,61E-01	9,66E-01	NS
MCP-3	0,03	9,69E-01	9,69E-01	NS
