# Supplementary material for: Predictive biomarkers of rapidly developing insulin deficiency in children with type 1 diabetes
Source: BMJ Open Diabetes Res Care. 2024 Feb 27;12(1):e003924. doi: 10.1136/bmjdrc-2023-003924 (PMC10900379; doi:10.1136/bmjdrc-2023-003924)
Supplement: Supplementary data [file bmjdrc-2023-003924supp004.pdf]

Welch two-sided T-test

Assay	Diabetes	Healthy	estimate	conf.low	conf.high	p.value	Adj.p.value	Threshold
IL4	1,65	3,25	-1,59	-2,05	-1,13	3,44E-09	4,69E-07	Sign
BACH1	2,07	2,85	-0,78	-1,02	-0,54	5,27E-09	4,69E-07	Sign
IRAK4	1,57	2,54	-0,97	-1,28	-0,66	1,60E-08	9,29E-07	Sign
MASP1	2,36	3,04	-0,68	-0,89	-0,47	2,09E-08	9,29E-07	Sign
DAPP1	2,86	3,95	-1,09	-1,47	-0,70	2,09E-07	7,46E-06	Sign
CLEC4D	3,25	4,33	-1,08	-1,47	-0,69	3,20E-07	9,12E-06	Sign
ICA1	2,09	2,94	-0,85	-1,16	-0,55	3,59E-07	9,12E-06	Sign
TNFSF14	6,66	7,60	-0,93	-1,29	-0,58	1,04E-06	2,31E-05	Sign
HCLS1	4,91	5,65	-0,74	-1,03	-0,46	1,46E-06	2,89E-05	Sign
Gal-1	7,90	8,16	-0,26	-0,36	-0,15	3,51E-06	6,25E-05	Sign
HEXIM1	4,91	5,69	-0,78	-1,10	-0,46	5,50E-06	8,89E-05	Sign
CASP-8	7,05	8,11	-1,06	-1,49	-0,62	6,93E-06	9,48E-05	Sign
PPP1R9B	4,00	4,83	-0,83	-1,18	-0,49	6,58E-06	9,48E-05	Sign
PIK3AP1	4,32	5,08	-0,76	-1,08	-0,44	9,49E-06	1,21E-04	Sign
TRIM21	3,49	4,35	-0,86	-1,22	-0,49	1,03E-05	1,22E-04	Sign
IL8	8,24	10,50	-2,26	-3,25	-1,27	1,79E-05	1,87E-04	Sign
PRDX5	7,37	7,99	-0,62	-0,89	-0,35	1,75E-05	1,87E-04	Sign
DCN	5,73	5,92	-0,19	-0,28	-0,10	3,84E-05	3,79E-04	Sign
GZMH	5,68	6,68	-1,00	-1,46	-0,54	4,60E-05	4,31E-04	Sign
CLEC6A	2,88	3,39	-0,50	-0,74	-0,27	4,94E-05	4,39E-04	Sign
LAP TGF-beta-1	3,21	3,72	-0,51	-0,74	-0,27	6,25E-05	5,30E-04	Sign
CCL23	11,49	11,88	-0,39	-0,58	-0,20	9,19E-05	7,43E-04	Sign
IRAK1	2,79	3,45	-0,66	-0,99	-0,33	1,70E-04	1,23E-03	Sign
GLB1	2,72	3,20	-0,48	-0,72	-0,24	1,68E-04	1,23E-03	Sign
ANG-1	10,82	11,23	-0,42	-0,63	-0,21	1,72E-04	1,23E-03	Sign
DFFA	6,04	6,68	-0,64	-0,97	-0,32	1,83E-04	1,25E-03	Sign
EGLN1	1,64	2,08	-0,45	-0,67	-0,22	2,01E-04	1,32E-03	Sign
PRDX1	2,59	3,20	-0,61	-0,92	-0,30	2,28E-04	1,45E-03	Sign
SIT1	3,25	3,89	-0,64	-0,97	-0,31	2,44E-04	1,50E-03	Sign
HGF	8,45	8,78	-0,33	-0,51	-0,16	3,28E-04	1,88E-03	Sign
CXCL12	1,46	1,64	-0,19	-0,29	-0,09	3,22E-04	1,88E-03	Sign
TREM1	-0,40	0,06	-0,46	-0,71	-0,21	3,45E-04	1,92E-03	Sign
PSIP1	2,23	2,99	-0,75	-1,16	-0,35	3,73E-04	1,95E-03	Sign
SRPK2	1,05	1,58	-0,53	-0,82	-0,25	3,69E-04	1,95E-03	Sign
CLEC4A	4,76	5,13	-0,38	-0,58	-0,17	4,88E-04	2,48E-03	Sign
SPRY2	2,63	3,20	-0,57	-0,88	-0,25	5,58E-04	2,76E-03	Sign
ADA	4,83	5,16	-0,33	-0,51	-0,15	6,02E-04	2,90E-03	Sign
TRIM5	4,12	4,83	-0,71	-1,11	-0,31	6,58E-04	3,08E-03	Sign
ARG1	3,93	4,63	-0,70	-1,10	-0,31	6,81E-04	3,11E-03	Sign
SH2D1A	2,67	3,34	-0,67	-1,04	-0,29	7,17E-04	3,19E-03	Sign
EDAR	3,67	4,39	-0,71	-1,12	-0,31	7,97E-04	3,46E-03	Sign
HSD11B1	3,14	3,43	-0,30	-0,46	-0,13	8,40E-04	3,56E-03	Sign

ITGA6	1,47	1,83	-0,36	-0,57	-0,15	8,95E-04	3,70E-03	Sign
TWEAK	10,43	10,73	-0,30	-0,47	-0,13	9,78E-04	3,96E-03	Sign
IRF9	2,85	3,35	-0,50	-0,79	-0,20	1,34E-03	5,32E-03	Sign
CD4	2,67	2,96	-0,28	-0,46	-0,11	1,52E-03	5,90E-03	Sign
EIF4G1	6,56	7,15	-0,58	-0,95	-0,22	1,90E-03	7,18E-03	Sign
BTN3A2	3,87	4,21	-0,34	-0,56	-0,13	2,01E-03	7,45E-03	Sign
PRKCQ	3,75	4,49	-0,74	-1,20	-0,28	2,05E-03	7,46E-03	Sign
DCTN1	5,17	5,74	-0,57	-0,93	-0,21	2,50E-03	8,90E-03	Sign
BIRC2	0,68	1,03	-0,35	-0,57	-0,12	2,76E-03	9,64E-03	Sign
FXYD5	1,47	1,87	-0,40	-0,66	-0,14	3,24E-03	1,11E-02	Sign
CXCL13	10,30	10,68	-0,38	-0,63	-0,13	3,79E-03	1,27E-02	Sign
CKAP4	5,39	5,72	-0,33	-0,55	-0,11	3,98E-03	1,31E-02	Sign
ITM2A	4,20	3,72	0,48	0,16	0,80	4,10E-03	1,33E-02	Sign
DDX58	3,35	4,05	-0,70	-1,18	-0,23	4,37E-03	1,39E-02	Sign
MMP12	7,18	6,77	0,41	0,13	0,69	4,47E-03	1,40E-02	Sign
CD40-L	8,97	9,57	-0,59	-1,01	-0,18	5,40E-03	1,60E-02	Sign
VEGFA	9,37	9,64	-0,27	-0,45	-0,08	5,26E-03	1,60E-02	Sign
KRT19	4,65	4,20	0,45	0,14	0,77	5,35E-03	1,60E-02	Sign
CLEC4G	3,34	3,64	-0,30	-0,51	-0,09	5,69E-03	1,66E-02	Sign
ICOSLG	6,72	6,94	-0,22	-0,37	-0,06	6,21E-03	1,75E-02	Sign
IL13	1,25	0,70	0,55	0,16	0,94	6,16E-03	1,75E-02	Sign
GZMB	4,44	4,93	-0,49	-0,84	-0,14	6,37E-03	1,77E-02	Sign
ZBTB16	3,97	4,55	-0,57	-0,99	-0,16	7,39E-03	1,99E-02	Sign
TANK	2,58	2,95	-0,37	-0,64	-0,10	7,49E-03	1,99E-02	Sign
IL12	8,59	8,27	0,31	0,09	0,54	7,39E-03	1,99E-02	Sign
IL7	5,72	6,10	-0,38	-0,67	-0,10	8,07E-03	2,10E-02	Sign
FGF2	0,58	0,77	-0,20	-0,34	-0,05	8,19E-03	2,10E-02	Sign
TNFRSF21	9,32	9,18	0,15	0,04	0,25	8,36E-03	2,10E-02	Sign
IL12RB1	3,03	2,79	0,24	0,06	0,42	8,36E-03	2,10E-02	Sign
EGF	11,34	11,75	-0,42	-0,73	-0,11	9,39E-03	2,32E-02	Sign
PDGF subunit B	12,63	12,77	-0,13	-0,24	-0,03	1,07E-02	2,61E-02	Sign
CCL19	11,27	10,99	0,28	0,07	0,49	1,11E-02	2,66E-02	Sign
CD83	4,43	4,22	0,21	0,05	0,38	1,16E-02	2,75E-02	Sign
CCL20	8,72	8,28	0,44	0,09	0,78	1,49E-02	3,50E-02	Sign
NCR1	3,09	3,35	-0,26	-0,47	-0,05	1,61E-02	3,72E-02	Sign
ADGRG1	1,67	1,84	-0,16	-0,30	-0,03	1,69E-02	3,85E-02	Sign
IL10	4,59	4,30	0,29	0,05	0,53	1,77E-02	3,99E-02	Sign
NF2	-1,20	-0,76	-0,43	-0,79	-0,07	2,01E-02	4,47E-02	Sign
AREG	3,00	3,43	-0,42	-0,78	-0,07	2,05E-02	4,51E-02	Sign
CCL4	9,25	9,83	-0,58	-1,09	-0,07	2,70E-02	5,86E-02	NS
ANGPT2	6,67	6,89	-0,22	-0,42	-0,02	2,91E-02	6,19E-02	NS
MGMT	4,91	5,31	-0,40	-0,75	-0,04	2,94E-02	6,19E-02	NS
CCL3	7,74	8,55	-0,81	-1,54	-0,08	2,96E-02	6,19E-02	NS
VEGFR-2	8,24	8,37	-0,12	-0,24	-0,01	3,12E-02	6,43E-02	NS
IL33	1,27	1,37	-0,10	-0,19	-0,01	3,14E-02	6,43E-02	NS
CDSN	3,34	3,55	-0,22	-0,42	-0,02	3,18E-02	6,44E-02	NS

TIE2	9,16	9,29	-0,13	-0,25	-0,01	3,57E-02	7,14E-02	NS
LY75	3,36	3,55	-0,19	-0,37	-0,01	3,64E-02	7,21E-02	NS
LAMP3	4,48	4,73	-0,25	-0,49	-0,01	4,12E-02	8,06E-02	NS
HO-1	13,03	13,23	-0,19	-0,38	0,00	4,48E-02	8,66E-02	NS
CXCL5	13,27	13,61	-0,35	-0,70	0,00	4,85E-02	9,28E-02	NS
CLEC7A	3,62	3,87	-0,25	-0,50	0,00	5,03E-02	9,53E-02	NS
IFNLR1	1,12	0,87	0,25	0,00	0,50	5,10E-02	9,56E-02	NS
IL6	3,83	4,44	-0,61	-1,23	0,01	5,55E-02	1,03E-01	NS
GZMA	6,83	7,01	-0,18	-0,36	0,01	5,78E-02	1,05E-01	NS
LILRB4	3,55	3,75	-0,19	-0,39	0,01	5,80E-02	1,05E-01	NS
IL6	2,83	3,42	-0,59	-1,21	0,03	6,21E-02	1,12E-01	NS
SH2B3	4,25	4,60	-0,34	-0,72	0,03	6,95E-02	1,24E-01	NS
FCRL3	2,27	2,47	-0,19	-0,41	0,02	7,34E-02	1,29E-01	NS
PLXNA4	8,05	8,44	-0,39	-0,85	0,07	9,67E-02	1,69E-01	NS
CCL17	10,41	10,68	-0,28	-0,61	0,06	1,06E-01	1,84E-01	NS
PTN	0,73	0,95	-0,23	-0,51	0,06	1,15E-01	1,96E-01	NS
CD8A	11,35	11,17	0,18	-0,04	0,40	1,15E-01	1,96E-01	NS
CXCL1	11,23	11,46	-0,23	-0,51	0,06	1,20E-01	2,02E-01	NS
HNMT	9,48	9,70	-0,22	-0,50	0,06	1,29E-01	2,14E-01	NS
CD5	6,80	6,97	-0,17	-0,39	0,05	1,34E-01	2,19E-01	NS
MCP-1	12,28	12,16	0,12	-0,04	0,28	1,34E-01	2,19E-01	NS
IL-1 alpha	-0,83	-1,05	0,22	-0,07	0,51	1,36E-01	2,21E-01	NS
TNFRSF12A	6,94	7,06	-0,12	-0,29	0,05	1,50E-01	2,39E-01	NS
MIC-A/B	4,87	5,44	-0,57	-1,36	0,21	1,50E-01	2,39E-01	NS
FAM3B	5,06	5,23	-0,17	-0,41	0,07	1,54E-01	2,43E-01	NS
TRAF2	3,96	4,22	-0,26	-0,63	0,11	1,63E-01	2,54E-01	NS
FCRL6	4,19	4,39	-0,20	-0,49	0,09	1,77E-01	2,75E-01	NS
CXCL12	2,35	2,43	-0,08	-0,20	0,04	1,80E-01	2,76E-01	NS
PDCD1	5,41	5,25	0,17	-0,08	0,41	1,88E-01	2,86E-01	NS
MCP-4	12,06	11,88	0,18	-0,10	0,47	2,05E-01	3,09E-01	NS
FGF2	1,21	1,33	-0,12	-0,31	0,07	2,11E-01	3,15E-01	NS
IL10	4,53	4,35	0,19	-0,11	0,48	2,16E-01	3,20E-01	NS
CD27	9,37	9,29	0,09	-0,05	0,23	2,18E-01	3,20E-01	NS
CD70	4,34	4,23	0,11	-0,07	0,28	2,20E-01	3,20E-01	NS
CLEC4C	5,15	5,30	-0,15	-0,40	0,09	2,22E-01	3,21E-01	NS
IL2	0,41	0,53	-0,12	-0,31	0,07	2,26E-01	3,24E-01	NS
KLRD1	7,21	7,38	-0,16	-0,44	0,11	2,37E-01	3,37E-01	NS
PRDX3	1,43	1,63	-0,20	-0,54	0,14	2,47E-01	3,49E-01	NS
IL12RB1	2,52	2,42	0,10	-0,07	0,28	2,52E-01	3,54E-01	NS
CD244	7,98	7,88	0,09	-0,08	0,26	2,85E-01	3,96E-01	NS
ITGB6	3,50	3,60	-0,10	-0,28	0,09	2,95E-01	4,07E-01	NS
NOS3	1,67	1,56	0,11	-0,10	0,32	3,14E-01	4,30E-01	NS
TNFRSF4	5,82	5,71	0,11	-0,10	0,32	3,16E-01	4,30E-01	NS
CCL11	8,79	8,87	-0,08	-0,25	0,09	3,32E-01	4,47E-01	NS
MILR1	4,66	4,77	-0,11	-0,35	0,12	3,44E-01	4,60E-01	NS
Gal-9	9,20	9,14	0,06	-0,07	0,19	3,52E-01	4,63E-01	NS

CNTNAP2	2,47	2,56	-0,09	-0,27	0,10	3,53E-01	4,63E-01	NS
TNFRSF9	8,09	8,00	0,09	-0,11	0,29	3,54E-01	4,63E-01	NS
GALNT3	2,28	2,39	-0,10	-0,32	0,12	3,59E-01	4,66E-01	NS
NCR1	4,63	4,55	0,08	-0,10	0,26	3,70E-01	4,77E-01	NS
IL18	10,47	10,36	0,11	-0,13	0,34	3,75E-01	4,80E-01	NS
CD83	4,25	4,16	0,09	-0,12	0,29	4,03E-01	5,09E-01	NS
PD-L1	6,42	6,35	0,08	-0,10	0,26	4,03E-01	5,09E-01	NS
MCP-2	8,98	9,11	-0,13	-0,45	0,19	4,14E-01	5,19E-01	NS
PADI2	1,56	1,73	-0,17	-0,60	0,25	4,18E-01	5,20E-01	NS
PGF	9,30	9,35	-0,05	-0,20	0,09	4,62E-01	5,69E-01	NS
LAG3	2,81	2,87	-0,06	-0,21	0,10	4,63E-01	5,69E-01	NS
IL5	3,48	3,72	-0,25	-0,97	0,48	5,04E-01	6,14E-01	NS
CXCL10	9,31	9,42	-0,11	-0,44	0,22	5,11E-01	6,19E-01	NS
TPSAB1	4,38	4,28	0,11	-0,23	0,45	5,29E-01	6,36E-01	NS
CD40	13,50	13,57	-0,07	-0,29	0,15	5,40E-01	6,46E-01	NS
KLRD1	7,24	7,17	0,07	-0,15	0,28	5,49E-01	6,48E-01	NS
KPNA1	1,47	1,27	0,20	-0,46	0,86	5,50E-01	6,48E-01	NS
ARNT	1,57	1,74	-0,17	-0,74	0,40	5,54E-01	6,48E-01	NS
JUN	1,40	1,24	0,17	-0,40	0,73	5,57E-01	6,48E-01	NS
DCBLD2	9,10	9,03	0,07	-0,17	0,31	5,70E-01	6,55E-01	NS
CXADR	2,08	2,14	-0,05	-0,23	0,13	5,72E-01	6,55E-01	NS
NFATC3	1,22	1,16	0,07	-0,17	0,31	5,74E-01	6,55E-01	NS
DGKZ	0,32	0,38	-0,05	-0,24	0,14	5,83E-01	6,56E-01	NS
NTF4	2,16	2,09	0,06	-0,16	0,29	5,84E-01	6,56E-01	NS
DPP10	2,26	2,32	-0,06	-0,28	0,16	5,86E-01	6,56E-01	NS
CD28	1,80	1,86	-0,05	-0,27	0,16	6,18E-01	6,88E-01	NS
ITGA11	2,94	2,99	-0,05	-0,25	0,15	6,29E-01	6,95E-01	NS
PTH1R	2,84	2,90	-0,06	-0,31	0,19	6,33E-01	6,95E-01	NS
STC1	6,53	6,47	0,06	-0,20	0,32	6,43E-01	6,99E-01	NS
TRAIL	9,12	9,08	0,04	-0,12	0,19	6,47E-01	6,99E-01	NS
CSF-1	9,44	9,47	-0,02	-0,13	0,08	6,48E-01	6,99E-01	NS
MMP7	12,07	12,10	-0,02	-0,15	0,11	7,28E-01	7,80E-01	NS
FASLG	8,04	8,07	-0,03	-0,20	0,15	7,44E-01	7,93E-01	NS
CXCL11	11,87	11,91	-0,03	-0,27	0,20	7,77E-01	8,23E-01	NS
IL5	2,16	2,23	-0,07	-0,71	0,56	8,17E-01	8,61E-01	NS
LAMP3	5,80	5,77	0,02	-0,19	0,24	8,26E-01	8,65E-01	NS
CRTAM	6,07	6,09	-0,02	-0,24	0,20	8,47E-01	8,80E-01	NS
EIF5A	1,68	1,71	-0,03	-0,36	0,30	8,50E-01	8,80E-01	NS
CD28	3,32	3,33	-0,01	-0,21	0,18	8,82E-01	9,07E-01	NS
CAIX	5,22	5,21	0,01	-0,16	0,19	8,94E-01	9,14E-01	NS
CX3CL1	6,97	6,96	0,01	-0,15	0,17	9,16E-01	9,32E-01	NS
PD-L2	3,75	3,74	0,01	-0,16	0,17	9,39E-01	9,50E-01	NS
CXCL9	8,47	8,48	-0,01	-0,30	0,29	9,65E-01	9,66E-01	NS
MCP-3	4,30	4,31	-0,01	-0,34	0,32	9,66E-01	9,66E-01	NS
